# Correlates of eating disorder pathology in Saudi Arabia: BMI and body dissatisfaction

**DOI:** 10.1186/s40337-022-00652-4

**Published:** 2022-08-24

**Authors:** Bernou Melisse, Matthijs Blankers, Edwin de Beurs, Eric F. van Furth

**Affiliations:** 1 Novarum Center for Eating Disorders & Obesity, Laan van de Helende Meesters 2, 1186 AM Amstelveen, The Netherlands; 2 Research Department, Arkin Mental Health Institute, Klaprozenweg 111, 1033 NN Amsterdam, The Netherlands; 3 Section Clinical Psychology, Leiden University, Wassenaarseweg 52, 2333 AK Leiden, The Netherlands; 4 Rivierduinen Eating Disorders Ursula, Sandifortdreef 19, 2333 ZZ Leiden, The Netherlands; 5 Department of Psychiatry, Leiden University Medical Center, Albinusdreef 2, 2333 ZA Leiden, The Netherlands

**Keywords:** Eating disorder pathology, BMI, Body dissatisfaction, Saudi Arabia

## Abstract

**Background:**

Saudi Arabia is undergoing rapid sociocultural changes, which may have led to an increase of body mass index and eating disorder pathology. The aim of this study is to investigate whether body dissatisfaction, self-esteem, having lived abroad, cultural orientation, perceived stress, media use, and socioeconomic status are correlates of eating disorder pathology with body mass index as a covariate. Additional aims are to investigate if cultural orientation is associated with symptomatology and if stress is a covariate in the association between eating disorder pathology and Western orientation.

**Method:**

Self-report measures were administered in a convenience Saudi community sample (*N* = 1225) between April 2017 and May 2018. Hierarchical multiple linear regression analyses with eating disorder pathology as dependent variable were performed to establish the associations among the variables.

**Results:**

After adjusting for the effect of BMI, only body dissatisfaction and eating disorder pathology were moderately associated. Eating disorder pathology and body dissatisfaction were more severe among Saudi citizens with a higher BMI.

**Discussion:**

Several explanations for the lack of associations of westernization, self-esteem, and stress with eating disorder pathology are reviewed and discussed. The majority of this convenience sample existed of young unmarried Saudi females of high socioeconomic status. Of the total sample, 35% displayed eating disorder pathology which may be a reflection of the high rates of excess weight.

**Supplementary Information:**

The online version contains supplementary material available at 10.1186/s40337-022-00652-4.

## Plain English summary

Eating disorders were assumed to be rare in Saudi Arabia, however, Saudi citizens report both, increased pressure to be thin and eating disorder pathology. Saudi Arabia is dealing with rapid sociocultural changes. Consequences of these sociocultural changes, which might be associated with eating disorder pathology, are an increase of BMI, body dissatisfaction, low self-esteem, westernization, perceived stress, media use, and socioeconomic status. In addition, the increase in BMI might strengthen the associations between eating disorder pathology and body dissatisfaction, low self-esteem, perceived stress, media use, and socioeconomic status. Alternative explanations might be that cultural values are associated with different eating disorder symptoms or that eating disorder pathology is associated with stress stemming from transformation that aims to empower women and modernize Saudi society. This study showed that only BMI and body dissatisfaction were associated with eating disorder pathology, and BMI was a covariate in the association between eating disorder pathology and body dissatisfaction. Preventative programs focused on avoidance of maladaptive weight loss strategies and the improvement of body satisfaction might be beneficial in Saudi Arabia.

**Supplementary Information:**

The online version contains supplementary material available at 10.1186/s40337-022-00652-4.

## Background

Eating disorders have historically been associated with Caucasian women in developed Western countries [1]. Therefore, eating disorders have been perceived as culture bound syndromes [2], and most research on eating disorders is conducted in Western countries [3]. However, cultural factors are essential to understand the etiology of eating disorders [4]. In Western countries, the main feature of eating disorders is the desire to be thin [1]. In Saudi Arabia traditional notions of beauty are different, with a curvy body ideal being associated with fertility and wealth. Thus, Saudi citizens are thought to be less likely to suffer from eating disorders [5]. However, though data are still inconclusive, the thin ideal seems on the rise in Saudi Arabia [6–8]. Recent studies have shown that eating disorders occur globally [9, 10] and eating disorders occur particularly in cultures in transition as they tend to adopt Western values [2, 11], illuminating the interplay between culture and psychopathology [12]. This is especially relevant to Saudi Arabia, as it is undergoing rapid sociocultural changes [6, 13].

Rapid sociocultural changes are often referred to as a combination of westernization, industrialization and globalization [1, 14, 15]. Westernization is defined as adoption of language, lifestyle, values and beliefs of Western cultures [14]. Western oriented Arabs display greater assimilation with Western countries than those who are more Arab oriented, resulting in Saudi citizens speaking English with their relatives and friends, and finding entertainment in malls rather than in a traditional souk [16]. In addition, industrialization comes along with an increased sedentary lifestyle, and a dietary shift towards Western types of foods, higher in salt, sugar and fat, all instrumental in the rise of excess weight [17, 18]. The prevalence of high BMI has increased from below 10% around 1980 to rates of 29–58%, and Saudi Arabia currently has one of the highest prevalence rates of excess weight [18–20]. Excess weight is associated with eating disorder pathology [21], as Saudi citizens with excess weight are known to be 2–3 times more likely to develop an eating disorder pathology than Saudi citizens without excess weight [22]. Furthermore, excess weight is associated with body dissatisfaction and low self-esteem [23]. Moreover, studies found that high BMI strengthens the association of eating disorder pathology with: body dissatisfaction, low self-esteem, perceived stress, and media use [17, 22, 24]. High BMI may serve as a covariate in the interplay between eating disorder pathology and its correlates.

Other consequences of westernization include an increase in media use, which may be associated with eating disorder pathology [3, 25]. Media use is defined as the use of internet, social media platforms, and streaming services [26]. Saudi citizens are extensive media users, internet access has increased over 100-fold in the last 16 years, and, on average, they spend 2.7 h daily watching streaming services, 50–100% more than in Western countries [27–29]. In addition, social media photos and videos often promote a more Western presentation of shape and weight, potentially resulting in increased thin ideal and consequently unhealthy dietary behavior [30]. Furthermore, various diets are promoted on social media [31]. However, the exact impact of westernization on the development of eating disorder pathology is still inconclusive. Some studies show that a Western orientation and having lived abroad, appeared to be risk factors for eating disorder pathology in several Gulf countries, and Gulf citizens who live in a Western country display more severe eating disorder pathology than their counterparts who still live in their country of origin [11, 22, 32–35]. In contrast, some studies show that internalization of Western values is weakly associated with eating disorder pathology in Saudi Arabia [36]. Finally, cultural orientation might be associated with symptomatology: individuals who are predominantly Arab orientated engage more in restrictive eating behaviors, while individuals who are more westernized display more binge eating behaviors [35, 37]. The role of religion may go both ways, as the Holy Quran supports restrictive eating stating that someone should fill its body 1/3 with air, 1/3 with water and 1/3 with food. Furthermore, restrictive eating during the holy month of Ramadan does not affect eating behavior [38–40]. On the other hand, social events are culturally accompanied by the intake of large amounts of food [41].

The contradictory results regarding westernization suggest that, rather than just westernization, conflicting cultural values related to the sociocultural, political and legal transformations that aim to empower women and modernize the relatively conservative Saudi society might cause stress [22, 42]. Maladaptive coping mechanisms to respond to stress may contribute to the development of eating disorders, as stress is associated with binge eating, restraint eating and overeating [43]. The role of perceived stress should therefore be taken into consideration as well, when examining correlates of eating disorder pathology [44]. Lastly, the sociocultural changes led to an increase in socioeconomic status (SES) of Saudi inhabitants since a free educational system is developed [6]. Consequently, level of education and employment rates of Saudi citizens increased [32, 45]. However, the exact impact of SES on eating disorder pathology is unclear: in Saudi Arabia SES appears to be associated with restrictive eating behavior [32], which is confirmed by studies conducted in Western countries. However, SES appears to be negatively associated with excess weight, whereas binge eating disorder appears unrelated to SES in Western societies [1, 46, 47]. Lastly, the increase in SES is associated with larger families and therefore an increase in youth. Median age in Saudi Arabia is 31.8 years and about 72% is aged between 15 and 64 years [48, 49]. The growth of the adolescent population is potentially associated with an increase in eating disorder pathology as the prevalence appears to increase among adolescent Saudi citizens [50–53].

In sum, as Saudi Arabia is undergoing rapid sociocultural changes, several risk factors for the development of eating disorder pathology may currently apply. Though studies have identified correlates of eating disorder pathology in Western countries, few have been undertaken in Saudi Arabia [26, 54]. Once correlates of eating disorder pathology are identified targeted preventative programs can be initiated. The aim of this cross-sectional explorative study was to examine whether body mass index (BMI), body dissatisfaction, self-esteem, cultural orientation, perceived stress, media use, and SES are correlates of eating disorder pathology in Saudi Arabia. Additional aim is to investigate if a high BMI is a covariate in these potential associations. It is hypothesized that, eating disorder pathology is positively associated with greater body dissatisfaction, low self-esteem, Western cultural orientation rather than Arab orientation, increased levels of perceived stress, more time spend on media use, and increased SES, and that a larger BMI serves as a covariate in these associations. An alternative hypothesis is that a greater level of stress is a covariate in the association between eating disorder pathology and Western orientation. An additional alternative hypothesis is that cultural orientation is associated with symptomatology, where Saudi citizens who are Arab oriented show more restrictive behavior and those with who are Western oriented more binge eating behavior.

## Methods

### Procedure

A convenience sample was recruited between April 2017 and May 2018 from students (Princess Noura bint Abdulrahman University (PNU), King Saud University, Sixth High School for Quran Memorization) in Riyadh, and through social media (Twitter, Facebook, Snapchat). Several sports facilities (NuYu gym, Sukoun yoga studio) in Riyadh, Dammam and Jeddah also shared a link among their members. Participants were also recruited through the first authors (BM) social network. Some of BMs students recruited participants through their personal network. Inclusion criteria were being a Saudi passport holder, literate and age ≥ 18 years old. The aim was to reach as many Saudi citizens as possible. As Saudi society is being perceived as a relatively culturally reclusive society [55], it is hard to access its citizens, and because eating disorder pathology has only been poorly studied, data collection involved targeting Saudi citizens in the country, which has resulted in a unique sample. All participants (*N* = 1229) were Saudi nationals, however four participants (3.2%) had ≥ 5% missing data with regard to measure items and were therefore excluded. Participants (*N* = 1225) were literate, and aged between 18–81 years old, mean age 23.6 (SD = 8.5) years (Table 1). Participants provided informed consent and completed anonymously an online self-report survey through Survey Monkey. Questions about the study could be sent by email to BM.

**Table 1:** Demographics

	*N*	*MD (S)*
Age	1225	23.6 (8.5)
18–25 years	919 (75.0%)	
26–40 years	228 (18.6%)	
41–65 years	70 (5.7%)	
66–81 years	3 (0.2%)	
Gender		
Unknown	11 (0.9%)	
Female	1048 (85.6%)	
Male	166 (13.6%)	
BMI	1225	25.2 (7.0)
Marital status	1225	
Unknown	1 (0.1%)	
Married	211 (17.2%)	
Unmarried	938 (76.5%)	
Divorced	75 (6.1%)	
Occupation/education	1225	
Unknown	15 (1.2%)	
High school	315 (25.7%)	
University in country of heritage	474 (38.7%)	
University in Arab country	25 (2.0%)	
University in Western country	6 (0.5%)	
Employed	182 (14.9%)	
Unemployed	127 (10.4%)	
Other	81 (6.6%)	
Self-report measures		
EDE-Q	1225	2.7 (1.4)
BSQ	1225	86.5 (36.3)
Rosenberg self-esteem	1220	27.0 (79.4)
ARMSA Western orientation	1222	1.1 (0.6)
ARMSA Arab orientation	1115	1.4 (0.5)
Perceived stress scale	1003	27.5 (71.8)
Eating disorder behaviors^†^, reported by *N*, (%)		
Objective binges	1078 (88.0%)	6.8 (3.4)
Self induced vomiting	392 (32.0%)	2.5 (6.0)
Laxative misuse	401 (32.7%)	3.0 (0.6)
Extensive exercise	967 (78.9%)	8.7 (9.8)

*ARMSA* Acculturation Scale for Mexican Americans, *BMI* Body Mass Index, *BSQ* Body Shape Questionnaire, *EDE-Q* Eating Disorder Examination-Questionnaire

^†^Frequency of eating disorder behaviors reported during the last 4 weeks

### Measures

#### Eating Disorder Examination-Questionnaire

The Saudi version of the Eating Disorder Examination-Questionnaire (EDE-Q) was used to assess eating disorder pathology during the last 28 days [19, 56]. The EDE-Q is a self-report instrument of 28 items, resulting in a global score indicating the severity of eating disorder pathology and additionally measures frequency of eating disorder behaviors such as binge eating episodes. Severity of eating disorder pathology was measured on a 7 point- Likert scale (0: feature was absent, to 6: feature was markedly present or present every day) [56]. The EDE-Q was translated to Arabic and some cultural adjustments were made in the Saudi EDE-Q e.g., communal changing rooms and pools are rare and were replaced by gyms and weddings, as further explained in Melisse et al. (2021) [19]. The Saudi version has good psychometric properties [19]. However, the factor structure with four subscales has not been confirmed with factor analysis. Therefore, only the EDE-Q global score was used. The Saudi EDE-Q has a Cronbach’s α of 0.89, clinical cut-of is 2.93 [19]. Cronbach’s α in this study was 0.90.

#### Body Shape Questionnaire

The Body Shape Questionnaire (BSQ), measures the severity of body dissatisfaction, such as fear of gaining weight, desire to lose weight and physical- appearance related self-devaluation during the last 28 days. The BSQ is a self-report measure of 34 items on a 6 point- Likert scale (1: never, to 6: always) [57]. The overall score is the total of all items and can range between 34 and 204, higher scores indicate greater body dissatisfaction. Scores ≥ 80 indicate mild concern, ≥ 111 moderate concern and ≥ 140 marked concern with shape. The BSQ has a Cronbach’s *α* of 0.96, a high test–retest reliability and good concurrent validity [58]. BM and a clinical psychology student of PNU slightly adapted an Arabic version of Mousa et al. (2010) with regard to the Arabic language [59]. In addition, one cultural adaptation was made in question 27: as females in Saudi Arabia share cars rather than travel by bus, “bus seat” was changed to “car seat”. Cronbach’s *α* in this study was 0.96.

#### Rosenberg self-esteem scale

The Rosenberg self-esteem scale measures self-esteem with 10 items representing statements with a 4-point Likert response scale (1: strongly disagree, to 4: strongly agree), five of these items (2, 5, 6, 8, 9) were reverse scored. Higher scores indicate higher self-esteem and sum scores can range from 10 to 40 [60]. Cronbach’s *α* of the Arabic version was 0.71, the instrument has a high test–retest reliability and good concurrent validity [61–63]. Cronbach’s *α* was 0.70 in the present study.

#### Acculturation Rating Scale for Mexican–Americans II

The revised version of the Acculturation Rating Scale for Mexican–Americans (ARMSA II) [64, 65] is a westernization survey that measures two unrelated dimensions: maintenance of the original culture and identification with the recently introduced culture, originally researched among Mexican immigrants. Western and Mexican orientation were each assessed in 12 behaviorally based questions on a four point Likert scale (1: never to 4: very often) for language, media, food and consumer goods. Total scores on both cultural orientation scales ranged between 0 and 36, scores on both dimensions can be used separately or deducted [64]. Adaptations were made similar to those of Stigler et al. (2010) who adapted the ARMSA II for use among Indians in their country of origin. In the adapted version Cronbach’s alphas were 0.84 for the Indian scale and 0.90 for the Western scale [66].

The ARMSA II was translated to Arabic by a native speaker, a clinical psychology student of PNU, with a parallel translation by a professional translator. Minor differences between both versions were discussed and resolved, and a backtranslation was performed. The ARMSA II was adjusted, resulting in an Arab orientation and Western orientation scale. The following cultural adaptations were made by BM: language preferences involving Arabic as mother tongue, English as Western language, media preferences as Arabic versus Western shows/films/music. With regard to food/restaurants, common Western coffee houses in Saudi Arabia such as Java Time, Starbucks and Dr. Cave, and common Arabic restaurants such as Najd Village and Isteraha (desert-camp), and traditional Saudi dishes such as kabsah, maqluba, dates, samboosa and dolmah, were added. Traditional markets were mentioned as souks, as were traditional clothes, jallabiyah (for women) and thoob (for men) respectively. Cronbach’s alphas were 0.82 for the Arab scale and 0.90 for the Western scale. Scores on these scales were weakly correlated (Pearson’s *r* = 0.27, *p* < 0.01).

#### Perceived Stress Scale

The Perceived Stress Scale (PSS) measures subjective feelings of stress on 10 items using a 5-point Likert scale (0: never to 4: very often) during the last month. Positively stated items (4, 5, 7, 8) were reverse scored, total score range is 0–40. Higher scores indicate more severe feelings of stress [67]. The Arabic version has Cronbach’s *α* of 0.80 and high test–retest reliability [68]. Cronbach’s *α* in the present study was 0.83.

#### Media use

Participants completed questions about usage (time and frequencies) of internet, social media and streaming services as described in Fardouly and Vartanian (2014) [69]. They also provided information about use of streaming services, provided in Additional file 1: Supplementary Table A. Internet and streaming services use were measured with a single item. Cronbach’s *α* was 0.80 for social media and 0.78 for overall media use in the present sample.

#### Body Mass Index

BMI was calculated as weight in kg/squared height in meters as reported in the EDE-Q. WHO guidelines were applied to classify BMI [70].

#### Socioeconomic status and demographic data

To estimate SES, questions about level of education, parental level of education, level of profession (e.g. manual, managerial) and parental level of profession were administered. Answers to these questions were administered as a Likert scale (e.g. level of profession 1: housewife/ unemployed, to 4: professional). Housewife was indicated as low SES since housewives in Saudi Arabia are generally lower educated and women of high SES are usually employed and can afford nannies, maids and drivers [32, 45, 71]. Each item received a numeric score of which all items together resulted in a total score, global scores were averaged item scores. Higher global scores represented higher SES. Demographic data such as gender, age, marital status, nationality, and history of living abroad were also requested from the participants.

### Statistical analysis

To test for multicollinearity between independent variables correlation analyses were performed. Differences in eating disorder pathology were examined by ANOVA for continuous variables (age) and chi-square for non-continuous variables (gender, marital status and education/profession). All data met the assumptions of normality, homoscedasticity, and linearity. A hierarchical multiple linear regression analysis, forward stepwise method was performed with body dissatisfaction, low self-esteem, having lived abroad for at least six months, cultural orientation, perceived stress, media use, and socioeconomic status as independent variables and eating disorder pathology as dependent variable. In step one, BMI was entered to control for its potential cofounding effect; in step 2, the independent indices were allowed to enter, to test whether they would predict additional variance in eating disorder pathology, as reflected in the EDE-Q total score, over BMI. In addition, a Bonferroni post-hoc analysis was performed. A one-way ANOVA was used to test if severity of eating disorder pathology and body dissatisfaction significantly differed among the various BMI groups. Data were analyzed in SPSS version 25.

### Ethical considerations

Ethical approval was given on May 7th, 2017 (17-0097), by the ethical review board of PNU in Riyadh, Saudi Arabia.

## Results

All participants (*N* = 1225) were Saudi nationals, literate, and aged between 18–81 years old, mean age 23.6 (SD = 8.5) years (Table 1). There were several differences between the study sample compared to the general Saudi population: females were overrepresented, 85.6% (*n* = 1048) versus 42.3%, the majority was single (76.5% vs. 33.0%) and a smaller percentage married (17.2% vs. 58.8%) [72]. The study sample was also more highly educated: most participants attended university (41.2% vs. 4.4%) [73], and 14.9% was employed, compared to 30.2% generally [74]. Participants were from all over Saudi Arabia (details available from BM), though most resided in the larger cities. Post-hoc calculated power, considering 13 predictors and an observed *R*^2^ of 0.60, *α* = 0.05 (2 sided) was 100%.

### Eating disorder pathology

There was no effect of age (*F*(62) = 4677.95; *p* = 0.510), gender (*χ*^2^(1) = 107.8; *p* = 0.83) or employment/education (*χ*^2^(6) = 718.18; *p* = 0.052) on the EDE-Q global score. There was an effect of marital status (*χ*^2^(2, 1220) = 209.55; *p* = 0.002). Married Saudi citizens had less eating disorder pathology than unmarried Saudi citizens. Of the participants, 34.8% (*n* = 426: 24.7%, *n* = 41 male, 36.3%, *n* = 380 female) had an EDE-Q score above the clinical cut-off of 2.93 [19]. Multiple comparisons (*F* = 160.39, *p* < 0.001) showed that, based on BMI, the average scores on the EDE-Q were different between Saudi citizens with a BMI < 18.5, a BMI between 18.5 and 24.9 and BMI between 25.0 and 29.9 (*p* < 0.001). EDE-Q scores did not differ between Saudi citizens with a BMI between 25.0 and 29.9 or 30.0 and 39.9 (*p* = 0.369) and between Saudi citizens with a BMI between 30.0 and 39.9 or BMI > 40 (*p* = 0.469). BSQ scores of Saudi citizens with BMI < 18.5 were comparable to those with BMI between 18.5 and 24.9 (*p* = 0.405), but differed for BMI between 25.0 and 29.9 (*p* < 0.001) (*F* = 149.52, *p* < 0.001). There were no differences in BSQ scores for BMI between 30.0 and 39.9 and BMI > 40 (*p* = 0.133) (Table 2). This suggests greater body dissatisfaction and eating disorder pathology among participants with a higher BMI.

**Table 2:** Means (and SDs) of EDE -Q and BSQ scores by five BMI groups and results of multiple comparisons of Tukey posthoc tests

BMI	1. BMI < 18.5		2. BMI between 18.5–24.9		3. BMI between 25.0–29.9		4. BMI between 30.0–39.9		5. BMI > 40	
	*n*	%	*n*	%	*n*	%	*n*	%	*n*	%
	131	11.0	562	47.3	281	23.7	281	14.6	40	3.4

	*M*	SD	*M*	SD	*M*	SD	*M*	SD	*M*	SD	Pairwise comparison *p* < 0.001
EDE-Q total score	1.6	1.3	2.4	1.3	3.3	1.1	3.6	1.1	3.9	3.9	1 < 2 < 3 = 4 = 5
BSQ score	53.7	19.6	75.3	30.0	102.3	31.4	112.4	35.6	132.8	34.7	1 = 2 < 3 = 4 = 5

*BMI* Body Mass Index, *BSQ* Body Shape Questionnaire, *EDE-Q* Eating Disorder Examination-Questionnaire

### Correlates of eating disorder pathology

Table 3 shows that there was no problematic multicollinearity between independent variables. Table 4 shows that in Step 1 of the hierarchical regression analysis, the association between BMI and eating disorder pathology was positive, statistically significant (*p* < 0.001) and substantial, accounting for 35.2% of the variance in eating disorder pathology. In Step 2, body dissatisfaction, self-esteem, having lived abroad, cultural orientation, perceived stress, media use, and socioeconomic status were allowed to enter. BMI and body dissatisfaction together explained 60% of the variance in eating disorder pathology. After adjusting for BMI, body dissatisfaction was moderately associated with eating disorder pathology, and explained 24.8% of the variance. BMI appeared to serve as a covariate. However, after entering body dissatisfaction no other variables contributed to explaining variance in eating disorder pathology. Together, all variables explained 64% of the variance (*R*^2^ = 0.64, *F* = 41.14, *p* < 0.001). Bonferroni post-hoc analysis did not reveal different results as all significance levels were *p* < 0.003.

**Table 3:** Pearson correlation matrix of independent variables

	BMI	Body dissatisfaction	Low self-esteem	Lived abroad	Western orientation	Arab orientation	Western–Arab orientation	Perceived stress	Streaming services	Internet	Social media	Overall media use	SES
BMI	1.00												
Body dissatisfaction	0.27**	1.00											
Low self-esteem	0.02	0.04	1.00										
Lived abroad	0.02	0.02	0.23**	1.00									
Western orientation	− 0.07	0.06	0.06	0.75	1.00								
Arab orientation	0.03	0.05	− 0.14	0.01	0.27**	1.00							
Western–Arab orientation	0.07	0.22	0.11	− 0.01	0.75	− 0.56	1.00						
Perceived stress	− 0.02	0.02	0.01	− 0.16	− 0.09	− 0.06	0.03	1.00					
Streaming services	0.45**	0.11**	− 0.04	− 0.36**	− 0.42**	0.13	− 0.20	− 0.03	1.00				
Internet	− 0.09	0.03	− 0.01	− 0.14*	− 0.05	0.12	0.02	0.04	− 0.35**	1.00			
Social media	0.02	0.02	0.04	− 0.23	0.06	0.04	0.001	0.04	− 0.36**	− 0.35**	1.00		
Overall media use	0.02	0.02	− 0.04	− 0.23	− 0.06	− 0.06	0.08	0.04	− 0.36**	0.99**	1*	1.00	
SES	0.04	0.15*	0.35**	0.31	− 0.20*	− 0.08	− 0.21	0.02	0.55**	0.18	0.38*	0.38*	1.00

*BMI* Body Mass Index, *SES* socio-economic status

*p < 0.05

**p < 0.001

**Table 4:** Multiple hierarchical regression with eating disorder pathology as dependent variable

	EDE-Q total score*						Correlations		95% confidence interval	
	*β*	*t*	*p*	*F*	*df*	Δ adjusted *R*^2^	Zero order	Part *r*	Lower bound	Upper bound
Step 1										
BMI	0.12	6.41	< 0.001	139.65	1224	0.35	0.60	0.60	0.10	0.14
Step 2										
BMI	0.07	2.99	< 0.001	156.9	1115	0.35	0.60*	0.38	0.05	0.12
Body dissatisfaction	0.58	9.26	< 0.001	143.8	1115	0.60	0.75*	0.50	0.42	0.74
Self-esteem	0.003	0.53	0.598	60.3	1190	NA	0.28	0.04	− 0.01	0.12
Lived abroad for ≥ 6 months	0.169	0.10	0.221	66.8	1223	NA	0.28*	0.09	− 0.104	0.44
Western orientation	0.003	0.01	0.991	0.1	1201	NA	− 0.02	0.001	− 0.50	0.51
Arab orientation	− 0.26	− 0.83	0.410	1.3	1201	NA	− 0.11	− 0.06	− 0.88	0.36
Western–Arab orientation	0.16	1.72	0.090	0.8	1201	NA	0.10	0.20	− 0.28	0.74
Perceived stress	0.02	1.04	0.302	0.8	1190	NA	0.03	0.08	− 0.02	0.06
Streaming services	− 0.02	− 0.53	0.598	0.3	1223	NA	− 0.02	− 0.01	− 0.07	0.04
Internet use	0.004	8.34	0.798	69.6	1223	NA	0.28*	− 0.02	− 0.05	0.07
Social media	− 0.02	0.61	0.796	68.5	1223	NA	0.28*	− 0.02	− 0.14	0.11
Media use total	0.003	0.61	0.542	68.7	1223	NA	0.28*	0.05	− 0.01	0.13
Socioeconomic status	− 0.11	− 0.48	0.631	80.2	1223	NA	0.38*	0.04	− 0.05	0.03

*BMI* Body Mass Index, *EDE-Q* Eating Disorder Examination-Questionnaire

*Significant at *p* < 0.001 level

Pearson zero-order correlations were statistically significant but weak between EDE-Q total score and having lived abroad (*r* = 0.28, *p* < 0.001), use of internet (*r* = 0.28, *p* < 0.001), social media (*r* = 0.28, *p* < 0.001), and SES (*r* = 0.38, *p* < 0.001). The associations with self-esteem (*p* = 0.598), Western orientation (*p* = 0.991), Arab orientation (*p* = 0.441), perceived stress (*p* = 0.365), and streaming services (*p* = 0.598) were not statistically significant. In addition, when both scales of cultural orientation were adjusted for the effect of the other (e.g. Western orientation deducted from Arab orientation) no association was found with eating disorder pathology (*p* = 0.090). The alternative hypothesis was not confirmed: perceived stress was not a covariate in the association between eating disorder pathology and westernization (*p* = 0.468). Linear regression analysis showed that there were no associations between Arab orientation and restrictive eating behavior as measured by the restraint scale of the EDE-Q (*p* = 0.297), neither between Western orientation and binge eating behavior as measured by the EDE-Q open ended question regarding number of binge eating episodes (*p* = 0.180).

## Discussion

The main findings of this explorative cross-sectional study are that BMI, body dissatisfaction, lived abroad, media use, and SES were associated with eating disorder pathology, as was single marital status. However, when these correlates were assessed together only body dissatisfaction and BMI appeared to be associated with eating disorder pathology. The results of this study regarding body dissatisfaction and BMI are comparable to other studies [17, 22]. Body dissatisfaction was moderately associated with eating disorder pathology after adjusting for the effect of BMI. In line with other studies [75–77], body dissatisfaction was the strongest correlate of eating disorder pathology among various other correlates, and Saudi citizens with a high BMI had both, greater severity of eating disorder pathology and body dissatisfaction. Around 35% of this Saudi community sample had an EDE-Q score above clinical cut-off. Therefore, the high prevalence of eating disorder pathology may reflect the high rates of excess weight and maladaptive strategies to lose weight. This is of concern, as around half of the Saudi population has a BMI above 25 [19]. Preventative programs focused on avoidance of maladaptive weight loss strategies and the improvement of body satisfaction might be beneficial.

This study did not find an association between westernization and EDE-Q scores. Other studies found associations between westernization and EDE-Q scores. Failure of this study to reproduce the association between eating disorder pathology and westernization may be explained by the fact that only small associations were found in some other studies [11, 36]. In addition, the rapid sociocultural changes have also been associated with elevated levels of industrialization [11, 16]. Industrialization has coincided with an increase in mental health problems including eating disorders [11, 78]. In addition, societies dealing with faster industrialization displayed greater risk for eating disorders [16, 52]. Furthermore, other studies found that cultural orientation was associated with specific eating disorder pathology [22, 24, 35, 37, 45]. This alternative hypothesis was also not confirmed in current study: Arab orientation and restrictive eating behavior, and Western orientation and binge eating behavior were not associated. In addition, other studies suggested the role of stress in the association between eating disorder pathology and westernization [44]. Stress was not a covariate in the association between westernization and eating disorder pathology in current study. Finally, results of current study were not in line with other studies who found associations between eating disorder pathology and self-esteem [23], use of internet, social media and streaming services [3, 25, 26], and SES [32]. However, since the association of working status (housewife versus employed) and SES of mothers appeared inconclusive [79] the analysis was repeated eliminating housewife from the composite SES score. This analysis did not yield different results. In addition, other studies found BMI to serve as a covariate in the associations of eating disorder pathology and perceived stress, media use, and low self-esteem [17, 22, 24]. None of these findings were confirmed in the present study. Explanation might be that in these other studies small associations were found, most samples involved teenagers rather than adults, while severity of eating disorder pathology was inversely related to age [80, 81].

Several study limitations should be taken into consideration. First, BMI was calculated on self-reported weight and height, and social desirability may have instigated participants to report lower weight than their actual weight [82]. Second, the composition of the convenience sample differed substantially from the general Saudi population regarding gender and education level. No effect on EDE-Q total score was found of age, gender and education/occupation. In addition, results of a convenience sample are less reliable than a clinical sample and potentially impacts generalizability [16, 19]. Third, the sample may also be biased by participants’ readiness to report their eating disorder pathology. Factors not measured may also have caused selection bias: respondents may have participated because they are more interested in health care, mental health care, or eating disorder pathology, or because they have more concerns regarding their body shape or eating behavior compared to the general population [19]. Consequently, the results should be interpreted with care, as generalizability to the overall Saudi population is limited. At the same time, this study does provide information regarding the association of eating disorder pathology, BMI and body dissatisfaction. Preventative programs can be offered to young unmarried Saudi citizens with body dissatisfaction, as selective prevention programs display larger effects than universal prevention programs [83–86]. In addition, improved knowledge is likely to increase the number of people seeking treatment and decrease the stigma of psychotherapy [16].

Although the Acculturation Rating Scale for Mexican–Americans II (ARMSA II) appeared reliable, there were small limitations to its internal validity: the ARMSA II originally measured acculturation among migrants in the USA rather than acculturation among populations dealing with sociocultural changes in their country of origin. However, the ARMSA II was validated and used in international studies. In addition, the overall frequency and time Saudi citizens spent on (social) media was examined. Some studies found that greater emotional investment, negative feedback seeking and social comparison were associated with eating disorder pathology rather than time spent or number of actions on (social) media [26, 87]. Finally, as only associations among the variables could be investigated, the cross-sectional nature of this study does not allow the drawing of inferences about causal relationships.

This study also has several strengths. It is one of the few large studies conducted in Saudi Arabia investigating eating disorder pathology and its correlates. The sample size was large, despite several challenges to collect data among Saudi citizens. Saudi Arabia is being perceived as a relatively culturally reclusive society [55]. Therefore, it was difficult to reach Saudi citizens, BM traveled the country to recruit participants. In addition, Saudi Arabia has to deal with stigma in relation to mental health problems, and a lack of knowledge about eating disorders [88–90]. Therefore, study participation in this study was not self-evident for Saudi citizens, as feelings of mistrust, lack of knowledge, and lack of awareness were barriers for study participation in other studies [91–93]. Since female adolescents were overrepresented in the sample results can be potentially be generalized to the eating disorder population as eating disorders are most prevalent among female adolescents [94, 95]. The hypothesis regarding the covariation role of acculturative stress among Saudi citizens with a Western orientation in the development of eating disorder pathology was not confirmed. However, though many studies assume the role of Western orientation and acculturative stress in the development of eating disorder pathology but do not actually measure both constructs, this study measured both. It is also the first study in Saudi Arabia to use a validated eating disorder screening instrument. Most other self-report measures used in this study also have strong psychometric properties and have been used in other Arab populations before. Furthermore, while most studies assume in- group homogeneity based on nationality, this study examined within-group variance by means of either Western or Arab orientation.

Future research should take into account the limitations of this research, in order to draw causal conclusions, experimental designs should be used. Relating specifically to the proposed implications of this study, causal studies could estimate whether body dissatisfaction and high BMI cause eating disorder pathology or whether they are a result of eating disorder pathology. These studies should use assessor based data regarding BMI to prevent bias [19]. Future research should also investigate if an extensive focus on body dissatisfaction in therapy is actually effective in terms of reduction in eating disorder pathology and relapse rate. In addition, the association between eating disorder pathology and media use should be investigated by emotional involvement rather than time spent or number of actions on (social) media [26, 87] and the use of a validated ARMSA II is recommended. Furthermore, examination of correlates and risk factors of eating disorder pathology is more reliable among clinical samples. However, eating disorders are rarely recognized and treated in Saudi clinics [16, 88]. Besides, Saudi citizens suffering from eating disorders generally seek psychiatric help only after suffering from somatic complaints such as diabetes, infertility, and kidney failure [19]. In addition, although Saudi citizens are an understudied population, a more balanced community sample regarding age, and educational level would potentially increase generalizability.

In conclusion, this explorative study contributes to the current state of knowledge on eating disorder pathology and its correlates in Saudi Arabia. It indicates that BMI is a covariate in the association between eating disorder pathology and body dissatisfaction, and that Saudi citizens with a higher BMI display more severe body dissatisfaction and eating disorder pathology.

## Supplementary Information

**Additional file 1. Appendix A. Supplementary Table A**. Media use.

## Data Availability

Upon reasonable request with BM.

## Abbreviations

ARMSA II: Acculturation Scale for Mexican Americans II
BMI: Body Mass Index
BSQ: Body Shape Questionnaire
EDE-Q: Eating Disorder Examination Questionnaire
PNU: Princess Noura bint Abdulrahman University
PSS: Perceived Stress Scale
SES: Socioeconomic status
WHO: World Health Organization
