# Supplementary material for: Correlates of eating disorder pathology in Saudi Arabia: BMI and body dissatisfaction
Source: J Eat Disord. 2022 Aug 24;10:126. doi: 10.1186/s40337-022-00652-4 (PMC9404570; doi:10.1186/s40337-022-00652-4)
Supplement: Supplementary file 1 — Additional file 1. Appendix A. Supplementary Table A. Media use. [file 40337_2022_652_MOESM1_ESM.docx]

|  | | | | | | |  |  |  |  |
| --- | --- | --- | --- | --- | --- | --- | --- | --- | --- | --- |
|  |  |  |  |  |  |  |  |  |  |  |

**Appendix A. Supplementary Table A** Media use

|  | Most common answer | % | *N* |
| --- | --- | --- | --- |
| On a typical day, how often in total do you check your social media accounts as Facebook, Snapchat, Instagram, twitter, MySpace etc. (even if you are logged on all day)? | Every few hours | 20.7 | 253 |
| Overall, how much time do you spend on your social media accounts on a typical day? | 4-6 hours | 24.4 | 298 |
| In general how often do you update your profile picture? | Sometimes | 26.9 | 329 |
| In general how often do you post a photo/ picture? | Almost never or never | 32.4 | 397 |
| In general how often do you send/receive private messages? | Sometimes | 20.9 | 256 |
| In general how often do you post a status update post a link to a news story, video, web site, etc. ? | Almost never or never | 27.8 | 338 |
| In general how often do you view friends’ photos that they’ve added of you ? | Sometimes | 26.0 | 318 |
| In general how often do you view friends’ photos of themselves? | Sometimes | 26.9 | 329 |
| In general how often do you view friends’ status updates ? | Sometimes | 21.6 | 264 |
| In general how often do you comment on friends’ photos? | Sometimes | 28.0 | 343 |
| In general how often do you comment on friends’ status updates ? | Sometimes | 23.9 | 293 |
| Overall, how much time do you spend on internet each week day (not work or homework related)? | 6 hours | 28.1 | 344 |
| Do you have access to streaming services such as OSN and Netflix, Amazon, HBO, Food Network, Fashion TV? | No | 35.7 | 437 |
| If yes, on average how many hours a day you spend watching those channels? | 0 | 68.6 | 840 |
